# Supplementary material for: Phosphoregulation of tropomyosin is crucial for actin cable turnover and division site placement
Source: J Cell Biol. 2019 Oct 9;218(11):3548–59. doi: 10.1083/jcb.201809089 (PMC6829654; doi:10.1083/jcb.201809089)
Supplement: Supplemental Materials (PDF) [file JCB_201809089_sm.pdf]

Supplemental material

Palani et al., https://doi.org/10.1083/jcb.201809089

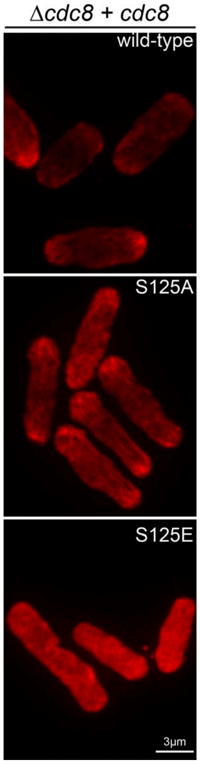

Figure S1. **Actin cable structures in *cdc8*, *cdc8*-S125A, and *cdc8*-S125E, stained with antibodies against Cdc8.** Shown are actin cable structures in *cdc8*, *cdc8-S125A*, and *cdc8-S125E*, stained with antibodies against Cdc8. Scale bar, 3 µm.

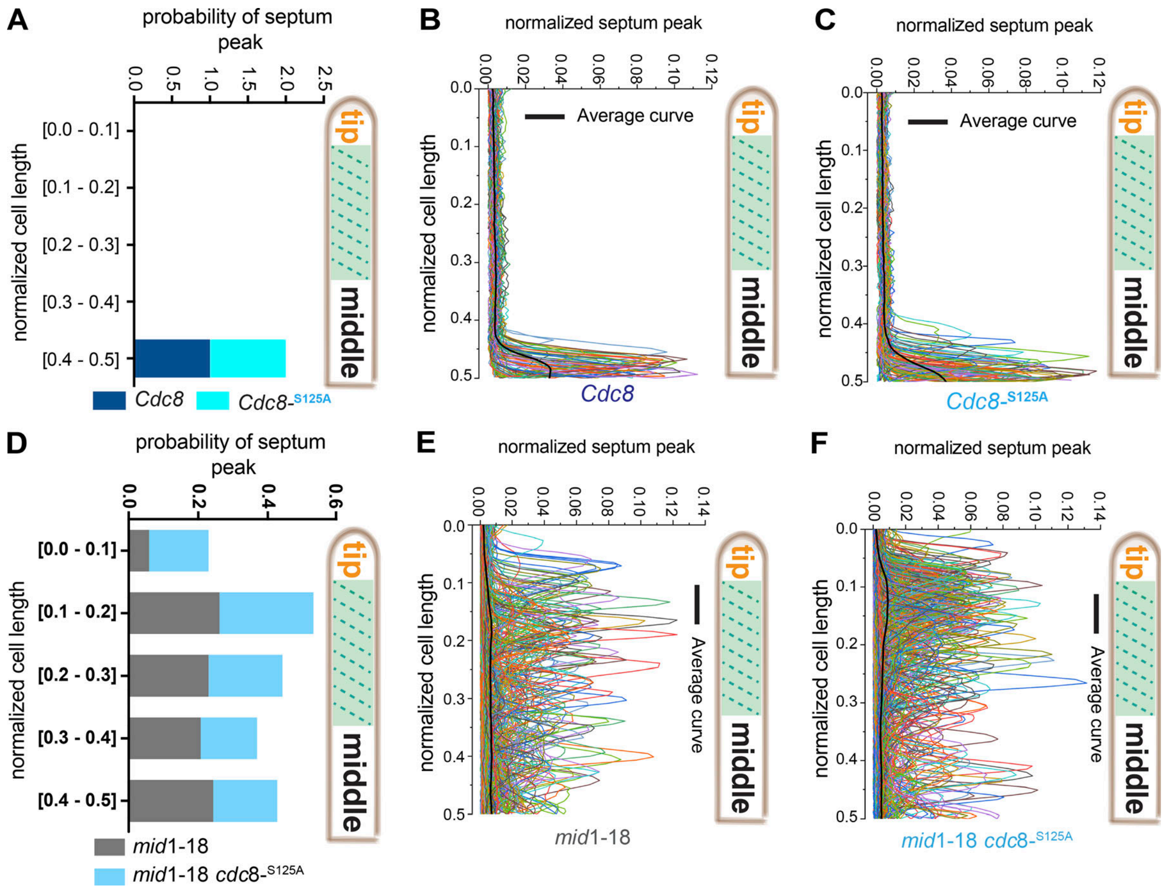

Figure S2. ***mid1*-18 *cdc8*-S125A mutant cells form tip septa at restrictive temperature.** **(A)** Histogram showing the relative occurrence of septum peak positions derived from individual scans shown in B and C. **(B and C)** Summary of individual line scans of the septum in Fig. 2 (E and F). **(D)** Histogram showing the relative occurrence of septum peak positions derived from individual scans shown in E and F. **(E and F)** Summary of individual line scans of the septum in Fig. 2 (E and F).

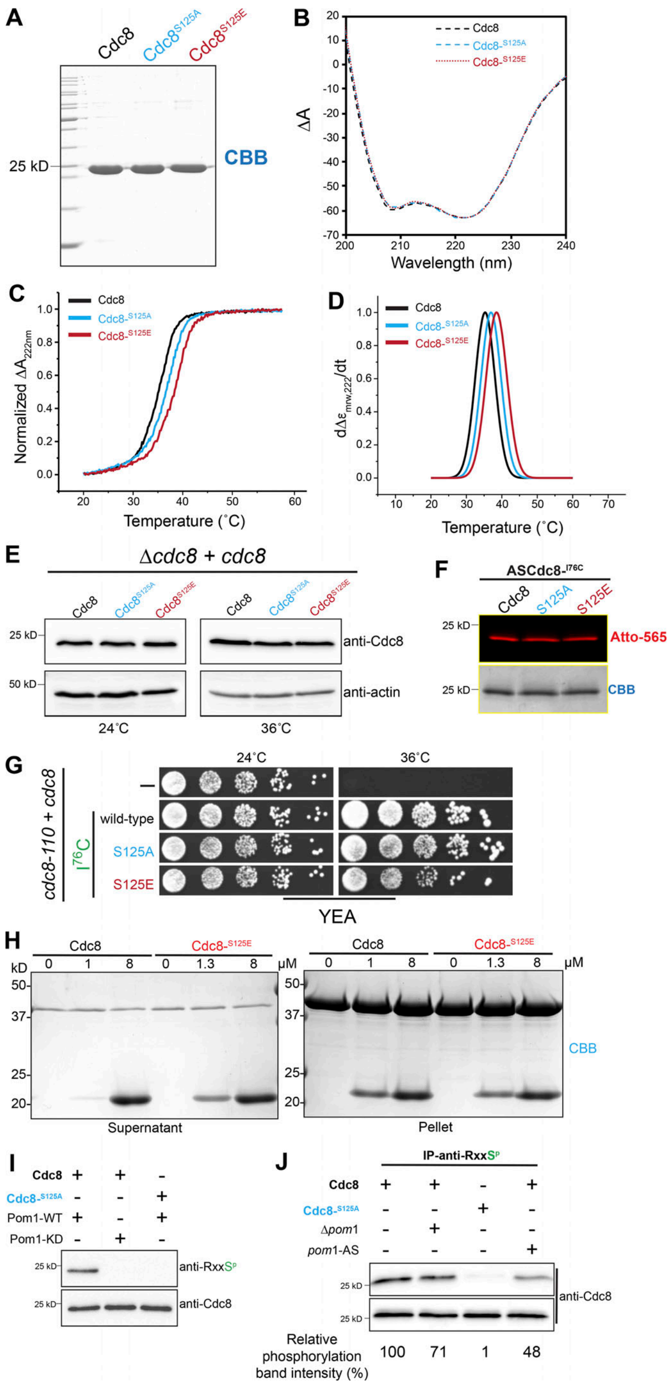

Figure S3. **Cdc8 S125A and Cdc8 S125E mutants are functional at higher temperatures; generation of functional fluorescent reporters for TIRF experiments. (A)** Coomassie blue–stained SDS-PAGE of purified amino-terminally acetylated WT and S125 mutants, which were used in CD and melting temperature experiments. **(B)** CD spectra of purified acetylated Cdc8 (black), S125A (blue), and S125E (red) Tpm^{Cdc8} proteins. **(C)** Normalized differential CD absorbance data for acetylated Tpm^{Cdc8} (black), acetylated Tpm^{Cdc8}–S125A (blue) and acetylated Tpm^{Cdc8}–S125E (red) at 222 nm. **(D)** First derivative plots at 222 nm of data described in C. **(E)** Mid-log phase WT cells and *cdc8*-S125A and *cdc8*-S125E were grown at 24°C or 36°C. Cells were lysed, and lysates were immunoblotted with antibodies against Cdc8 or actin (loading control). **(F)** Fluorescence and Coomassie blue–stained gels of purified acetylation mimicking version of ASCdc8 mutants (ASCdc8-I76C, ASCdc8-I76C-S125A, and ASCdc8-I76C-S125E) labeled with atto-565. **(G)** 10-fold serial dilutions of *cdc8+*, *cdc8*-S125A, and *cdc8*-S125E carrying either I76C in a *cdc8*-110 background were spotted onto YES plates and grown for 3 d at 24°C or 36°C. **(H)** Cosedimentation assay of actin-tropomyosin was performed using high-speed centrifugation. SDS-PAGE gel of supernatant stained with Coomassie blue. Each lane represents a pelleting experiment with a different concentration of ASCdc8 or ASCdc8-S125E. **(I)** In vitro kinase assay was performed by incubating recombinant GST-Pom1-WT and GST-Pom1-KD with ASCdc8 or ASCdc8-S125A. Proteins were run on SDS-PAGE, and phosphorylated sample was detected using anti-RxxSp antibody (top). One 10th of each Cdc8 protein input is shown as a control (bottom) using anti-cdc8 antibody. **(J)** Phosphorylated Cdc8 was immunoprecipitated from four different background strains (WT, *pom1Δ*, *cdc8*-S125A, and *pom1*-as1) using magnetic beads coupled with anti-RxxSp antibody. Immunoprecipitated samples were run on SDS-PAGE, and phosphorylated cdc8 was detected (top) using anti-cdc8 antibody. One 20th of each whole-cell extract protein input is shown as a control (bottom) using anti-cdc8 antibody.

Table S1. **List of strains used in this study**

**MBY192**	***ura4*-D18, *leu1*-32, h-**	**Lab stock**
MBY11063	*plys-cdc8* integrated in to lysine locus, *ura4cdc8Δ::NatMX6*. h+	This study
MBY11065	*plys-cdc8*-S125A integrated in to lysine locus, *ura4+ cdc8Δ::NatMX6*. h+	This study
MBY11067	*plys-cdc8*-S125E integrated in to lysine locus, *ura4+ cdc8Δ::NatMX6*. h+	This study
MBY11100	*mCherry-atb2::hph;rlc1-3GFP::kanMx6*; plys-cdc8 integrated in to lysine locus, *ura4+; cdc8Δ::NatMX6*.	This study
MBY11102	*mCherry-atb2::hph;rlc1-3GFP::kanMx6*; *plys-cdc8*-S125A integrated in to lysine locus, *ura4+; cdc8Δ::NatMX6*.	This study
MBY11104	*mCherry-atb2::hph;rlc1-3GFP::kanMx6*; *plys-cdc8*-S125E integrated in to lysine locus, *ura4+; cdc8Δ::NatMX6*.	This study
MBY11603	*plys*-empty integrated in to lysine locus, *ura4+* of MBY109 (cdc8-110 ade6-210 ura4-D18 leu1-32 h-)	This study
MBY11605	*plys*-cdc8-^{I76C} integrated in to lysine locus, *ura4+* of MBY109 (*cdc8-110 ade6-210 ura4-D18 leu1-32* h-)	This study
MBY11607	*plys*-cdc8-$^{S125A\_I76C}$ integrated in to lysine locus, *ura4+* of MBY109 (*cdc8-110 ade6-210 ura4-D18 leu1-32* h-)	This study
MBY11607	*plys*-cdc8-$^{S125E\_I76C}$ integrated in to lysine locus, *ura4+* of MBY109 (*cdc8-110 ade6-210 ura4-D18 leu1-32* h-)	This study
MBY12656	*leu1*-32, *ura4*-D18, *ade6*-21X, Δ*cdc8*::natMX6, *lys1*<<pLYS1U-*Pcdc8*:*cdc8*, *mid1*-18, mCherry:*atb2*:hph h?	This study
MBY12658	*leu1*-32, *ura4*-D18, *ade6*-21X, Δ*cdc8*::natMX6, lys1<<pLYS1U-*Pcdc8*:*cdc8*-^{S125A}, *mid1*-18, mCherry:*atb2*:hph h?	This study
MBY12655	*leu1*-32, *ura4*-D18, *ade6*-21X, Δ*cdc8*::natMX6, *lys1*<<pLYS1U-*Pcdc8*:*cdc8*, *mid1*-18, *rlc1*:3GFP:kanMX6, mCherry:*atb2*:hph h?	This study
MBY12726	*leu1*-32, *ura4*-D18, *ade6*-21X, Δ*cdc8*::natMX6, *lys1*<<pLYS1U-*Pcdc8*:*cdc8*-^{S125A},*mid1*-18, *rlc1*:3GFP:kanMX6, mCherry:*atb2*: hph h?	This study
MBY10425	*pLYS-empty* integrated in to LYSINE locus, *URA4+ of MBY109 (cdc8-110 ade6-210 ura4-D18 leu1-32 h-)*	This study
MBY10426	*pLYS-cdc8* integrated in to LYSINE locus, *URA4+ of MBY109 (cdc8-110 ade6-210 ura4-D18 leu1-32 h-)*	This study
MBY12739	*pLYS-cdc8-S5A [S45;S47;S50;S55 and S125]* integrated in to LYSINE locus, *URA4+* of MBY109 (*cdc8-110 ade6-210 ura4-D18 leu1-32 h-*)	This study
MBY12740	*pLYS-cdc8-S5E [S45;S47;S50;S55 and S125]* integrated in to LYSINE locus, *URA4+* of MBY109 (*cdc8-110 ade6-210 ura4-D18 leu1-32 h-*)	This study
MBY3184	*pom1::ura4+ leu1-32 h+*	This study
YSM563	*h+ pom1-as1 (T778G) ade6-M216 leu1-32 ura4-*	S. Martin laboratory (University of Lausanne, Lausanne, Switzerland)

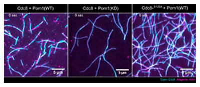

Video 1. **TIRF imaging of Cdc8 and Cdc8-S125A coated F-actin after addition of GST-Pom1-WT and GST-Pom1-KD.** Example image sequences of Cdc8-wt-Atto555 (cyan) decorated F-actin (Alexa488, magenta) after addition of Pom1(WT) (left) or Pom1(KD) (center) or of Cdc8-S125A-Atto555 (cyan) decorated F-actin (magenta) after addition of Pom1(WT) (right) at t = 0 s; 2 s per frame.

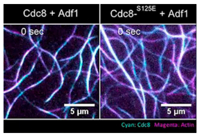

Video 2. **TIRF imaging of Cdc8 and Cdc8-S125E coated F-actin after addition of Adf1 (Cofilin).** Example image sequences of Cdc8-wt-Atto555 (cyan; left) or Cdc8-S125E-Atto555 (cyan; right) decorated F-actin (Alexa488, magenta) after addition of the actin severing factor Adf1 (cofilin) at t = 0 s; 2 s per frame.

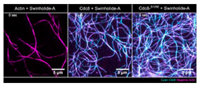

Video 3. **TIRF imaging of actin alone or Cdc8/Cdc8-S125E coated F-actin after addition of the actin-severing drug (Swinholide-A).** Example image sequences of F-actin (Alexa488, magenta) alone (left), Cdc8-wt-Atto555 (cyan; center,) or Cdc8-S125E-Atto555 (cyan; right) decorated F-actin after addition of the actin severing drug Swinholide-A at t = 0 s; 2 s per frame.
